# Changes in the posterior corneal surface after femtosecond laser-assisted lenticule intrastromal keratoplasty (LIKE) performed into a pocket (SMI-LIKE) or under a flap (FS-LIKE)

**DOI:** 10.1186/s40662-023-00337-2

**Published:** 2023-05-01

**Authors:** Shengtao Liu, Lanhui Yu, Yu Zhao, Xingtao Zhou

**Affiliations:** 1 Department of Ophthalmology and Optometry, Eye and ENT Hospital of Fudan University, Shanghai, China; 2 NHC Key Laboratory of Myopia, Chinese Academy of Medical Sciences, Fudan University, Shanghai, China; 3 Shanghai Research Center of Ophthalmology and Optometry, Shanghai, China; 4 Shanghai Engineering Research Center of Laser and Autostereoscopic 3D for Vision Care (20DZ2255000), Shanghai, China; 5 Affiliated Eye Hospital of Nanchang University, Nanchang, China

**Keywords:** Hyperopia, Lenticule implantation, Femtosecond laser, Posterior corneal elevation

## Abstract

**Background:**

To compare the changes in posterior corneal surface after small-incision lenticule intrastromal keratoplasty (SMI-LIKE) and femtosecond laser-assisted lenticule intrastromal keratoplasty (FS-LIKE) for hyperopia correction.

**Methods:**

In this prospective comparative randomized study, 23 eyes with hyperopia were recruited. Eyes were categorized into two groups—SMI-LIKE group (11 eyes) and FS-LIKE group (12 eyes). Lenticules from myopia small incision lenticule extraction were implanted into a pocket (SMI-LIKE group) or at a depth of 100 µm under a flap (FS-LIKE group). Posterior corneal elevations in the center, mid-periphery, and periphery, as well as mean keratometry of the posterior corneal surface (Kmb) were measured using a Pentacam over a three-month follow-up.

**Results:**

All surgeries were completed successfully and no complications occurred. At one day postoperatively, there was a slight backward change with SMI-LIKE and a forward change with FS-LIKE in the central region of the posterior corneal elevation. Conversely, the peripheral area showed forward displacement in SMI-LIKE and an apparent backward change in FS-LIKE. The mid-peripheral regions manifested a backward change after the procedure throughout the entire follow-up in both groups. Kmb exhibited flattening at one month postoperatively and subsequently returned to its original level at three months after SMI-LIKE while in FS-LIKE, Kmb steepened after lenticule implantation with a significant change noted at one day postoperatively (*P* = 0.001).

**Conclusions:**

Posterior corneal surface after SMI-LIKE and FS-LIKE exhibited different change patterns in various corneal regions, with the most prominent change occurring at one day postoperatively during the three-month follow-up.

*Trial registration*: Chinese Clinical Trial Registry: ChiCTR-ONC-16008300. Registered on Apr 18th, 2016. http://www.chictr.org.cn/edit.aspx?pid=14090&htm=4

## Background

Hyperopia is a common type of refractive error that can be corrected using contact lenses, spectacles, or surgical techniques. Surgical management for hyperopia correction includes photorefractive keratectomy, laser in situ keratomileusis (LASIK), and small incision lenticule extraction (SMILE) to name a few [1]. However, compared to laser surgery for myopia correction, low predictability, poor stability, and high postoperative regression are common unresolved issues in hyperopia correction [2–4].

Recently, lenticule intrastromal keratoplasty using convex-shaped lenticules derived for hyperopia correction has evolved from myopia SMILE [5]. During the procedure, a myopic lenticule is implanted into the corneal stroma to steepen the center and flatten the periphery, and thus improve the patient’s uncorrected distance visual acuity (UDVA) and near vision [6]. As a corneal tissue additive surgery, lenticule keratoplasty shows potential advantages over traditional surgery for hyperopia correction e.g., requiring no corneal tissue ablation or extraction, facilitating a high degree of hyperopia correction and, the shape of the cornea is more natural postoperatively. Moreover, the surgery is reversible as the implanted lenticule can be replaced in case of complications. Currently, two alternatives for lenticule intrastromal implantation have been proposed. In small-incision lenticule intrastromal keratoplasty (SMI-LIKE), the lenticule is implanted into an intrastromal pocket via a small incision, whereas in femtosecond laser-assisted lenticule intrastromal keratoplasty (FS-LIKE), the myopia lenticule is transplanted beneath the femtosecond laser flap [7]. Researchers found that both methods result in low rejection rates for hyperopia treatment [8]. However, residual refractive errors still exist in most patients, and the efficacy and predictability of these two techniques need to be improved.

Apart from the reshaped anterior corneal surface in lenticule keratoplasty, several other factors are considered to affect postoperative refraction [9–11]. The posterior corneal surface is one such factor, and thus is theoretically free of the surgical impact of the procedure. Among a few parameters, posterior corneal elevation is a highly sensitive indicator of posterior corneal stability. To the best of our knowledge, this study is the first to investigate changes in posterior corneal surface following femtosecond laser-assisted lenticule implantation.

Here, we compared the short-term effects on the stability of the posterior corneal surface after performing SMI-LIKE and FS-LIKE for hyperopia lenticule implantation.

## Methods

### Patients

In accordance with the tenets of the Declaration of Helsinki, the Ethics Committee of the Affiliated Eye Hospital of Nanchang University Review Board (Jiangxi, China) approved the study protocol (KJ2008-10). All patients fully understood the treatment and provided written informed consent before study participation.

In this prospective, comparative study, 23 eyes of 18 patients with hyperopia at the Affiliated Eye Hospital of Nanchang University, China were recruited. The patients had no ocular diseases other than refractive error and met the following inclusion criteria: presence of hyperopia in ≥ 1 eye, age > 18 years, and a strong willingness to correct hyperopia. All patients underwent a systematic preoperative ophthalmologic examination, including slit-lamp examination, measurement of UDVA, corrected distance visual acuity (CDVA), intraocular pressure, and Pentacam high resolution (HR) imaging.

Eyes were randomly categorized into two groups—SMI-LIKE group (11 eyes of 9 patients) and FS-LIKE group (12 eyes of 9 patients). In the SMI-LIKE group, allogenic lenticules extracted from myopia SMILE were implanted into a femtosecond laser made pocket. While in the FS-LIKE group, allogenic lenticules were positioned under a femtosecond laser flap. Posterior corneal elevation was measured using the Pentacam over a three-month follow-up period.

### Surgical techniques

The same experienced surgeon performed all procedures (SL). The VisuMax femtosecond laser system (Carl Zeiss Meditec AG, Germany) with a repetition rate of 500 kHz and pulse energy of 130 nJ was used to perform all surgeries. Track distance and spot distance was set at (i) 4.5 µm in the lenticule and cap and (ii) 2.0 µm in the lenticule side and cap side.

Before surgery, donor patients underwent a comprehensive blood test, including blood routine examination, liver and renal function tests, and screening for infectious diseases (human immunodeficiency virus, hepatitis B virus, hepatitis C virus and syphilis). SMILE procedures were performed on donors using the myopia treatment mode. The femtosecond laser settings were as follows: 120 µm intended cap thickness, 6.5–6.8 mm optical zone (lenticule diameter), 7.5 mm cap diameter; and a 2 mm side cut at 120°. The lenticule was manually extracted and prepared with caution for re-implantation. To avoid flipping the lenticule orientation, we marked the anterior surface of the lenticule at the incision position with a marker.

**Fig. 1:**
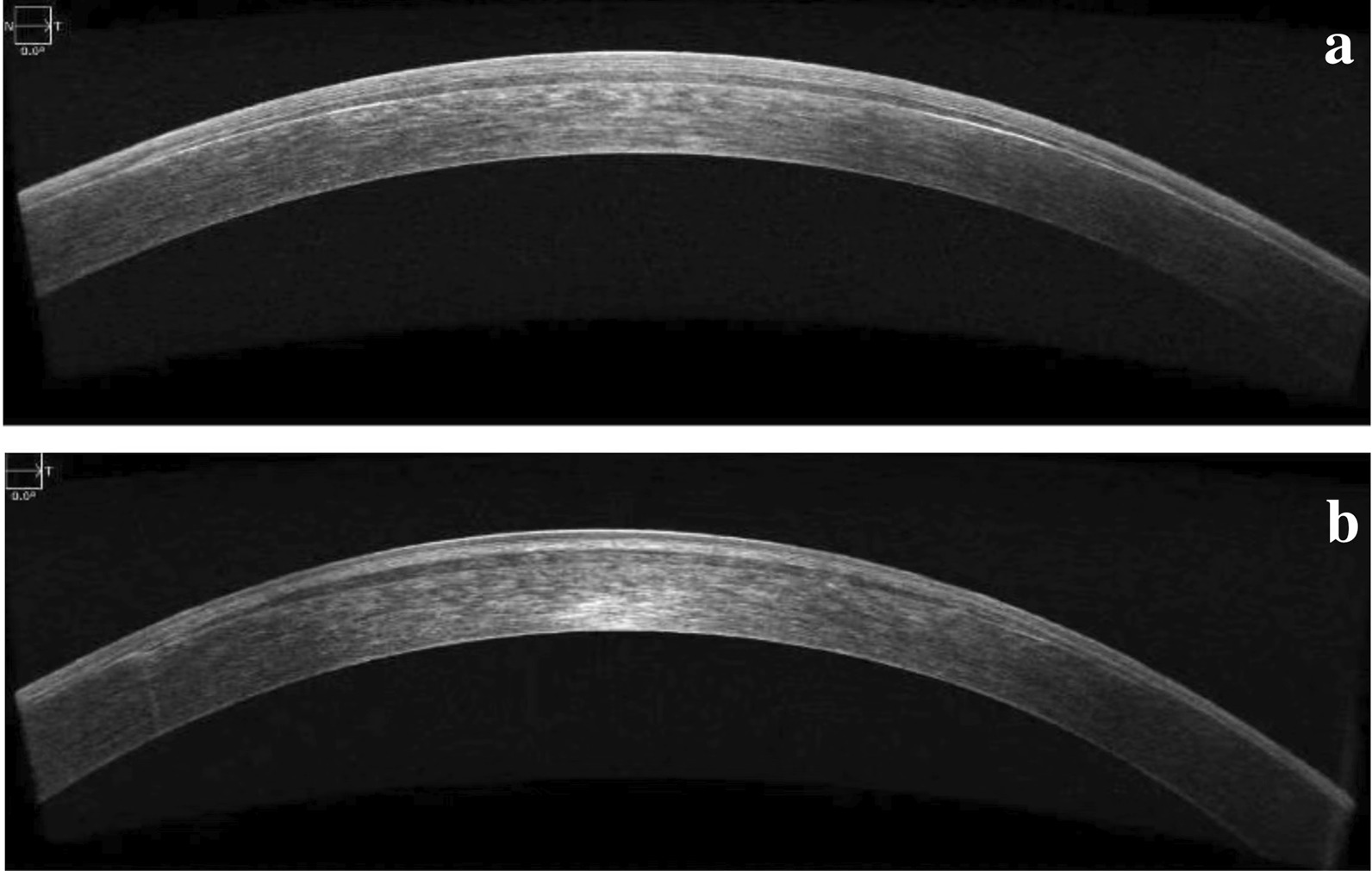
Corneal images of SMI-LIKE and FS-LIKE. **a** Anterior optical coherence tomography image of SMI-LIKE. **b** Anterior optical coherence tomography image of FS-LIKE. SMI-LIKE, small-incision lenticule intrastromal keratoplasty; FS-LIKE, femtosecond laser-assisted lenticule intrastromal keratoplasty

Lenticule implantation surgery was scheduled on the same day as the donor eye procedure. The diopter of the implanted lenticule was determined according to the spherical error of the subjective refraction of the recipient with an opposite sign. In the SMI-LIKE group, the stromal pocket was created using a flap license. The intended cap thickness was set at 100 µm and the diameter was 7.9 mm. A hinge length of 330° was used, thereby providing a 30° superior incision for lenticule implantation. Once laser scanning was completed, the surgeon inserted a spatula into the cornea and dissected the pocket plane. The extracted lenticule was then inserted into the pocket. In the FS-LIKE group, a flap of 100 µm and diameter of 7.9 mm was created instead of the cap. The hinge was located at 90°, with a length of 50°. After lifting the flap, the fresh donor lenticule was transferred directly onto the exposed stromal bed. The flap was replaced, and a bandage contact lens was placed over the cornea after surgery. For all surgeries, centration was accepted when the lenticular margin was concentric with the margin of the pocket or stromal bed. Representative images of SMI-LIKE and FS-LIKE are shown in Fig. 1.

Postoperative topical medication regimens were identical for each eye and consisted of 0.5% levofloxacin four times per day for seven days and prednisolone acetate ophthalmic suspension was applied topically four times daily for seven days. After the first week, the medication was changed to topical 0.1% fluorometholone four times daily for one month, and subsequently the application frequency was decreased to once a day for another month. The contact lens in the FS-LIKE group was removed one day postoperatively.

### Postoperative examination

Follow-up appointments were scheduled 1 day, 1 month, and 3 months after surgery. Postoperative examinations included Pentacam imaging examinations, slit-lamp examination, UDVA and CDVA measurement, spherical equivalent (SE) refraction, and intraocular pressure.

### Pentacam Scheimpflug imaging

All eyes were examined using the Pentacam imaging system (Oculus GmbH, Wetzlar, Germany). After attaining patient alignment, the device captured 25 images and automatically recorded 12,500 elevation points within 2 s. To avoid miscalculations of poor imaging, the quality of each measurement was shown in the specification window, and only results with “OK” statements were accepted. The examination was duplicated if the statement did not meet the requirements (marked as yellow or red). Only maps with at least 10 mm of corneal coverage and no deduced data in the central 9 mm zone were accepted.

### Data collection

**Fig. 2:**
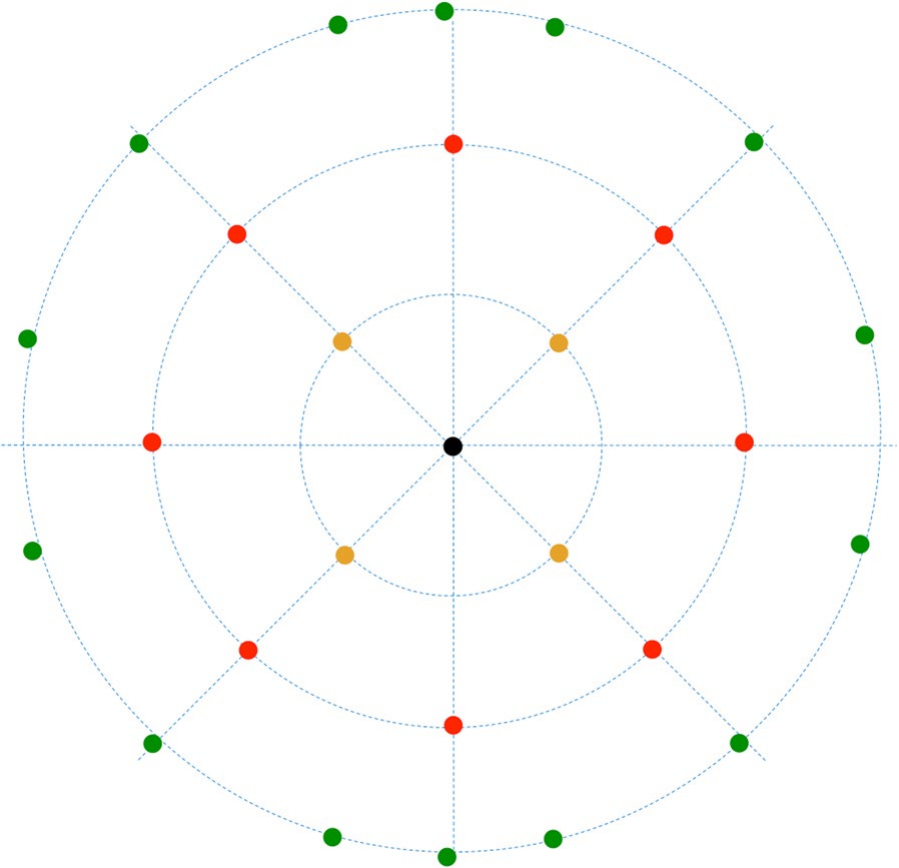
Overview of all the calculated values in Pentacam. Posterior central elevation (PCE, black dot); Mean value from 4 points in 2-mm diameter (PME-2 mm, yellow dot); Mean value from 8 points in 4-mm diameter (PME-4 mm, red dot); Mean value from 14 points in 6-mm diameter (PME-6 mm, green dot); Mean value from 13 points in the central 4-mm area (PCE-4 mm, black dot, yellow dot and red dot). PME, posterior mean elevation

Mean keratometry of the posterior corneal surface (Kmb) and elevation data were extracted directly from the Pentacam image. The reference best-fit sphere (BFS) was defined in the central 8.0 mm region of the preoperative data to be equal across images. For points above the reference, the values were positive; for points below, the values were negative. The calculated value of a single point was the posterior central elevation (PCE) above the BFS. The other 26 determined points in the central 6 mm zone were obtained as follows: 4 points at a distance of 1 mm from the center along the 45°, 135°, 225°, and 315° meridians (0° was defined as the horizontal semi-meridian on the right, and rotating counterclockwise in both eyes), 8 points at a 2 mm distance from the center at 0°, 45°, 90°, 135°, 180°, 225°, 270°, 315°, and 14 points at a distance of 3 mm from the center along 15°, 45°, 75°, 90°, 105°, 135°, 165°, 195°, 225°, 255°, 270°, 285°, 315°, and 345°. The posterior corneal elevation in the central 4 mm area (PCE-4 mm) and in various concentric circles (2 mm diameter, posterior mean elevation (PME)-2 mm; 4 mm diameter, PME-4 mm; 6 mm diameter, PME-6 mm) was calculated as the mean value from points in the corresponding area. Graphs of all the calculated values are displayed in Fig. 2. Changes in elevation were determined by subtracting the preoperative data from postoperative data (difference elevation map). The change in elevation was due to a shift in the posterior corneal surface. All data were recorded in an Excel spreadsheet (Microsoft Corp., Redmond, WA, USA) for further analysis.

### Statistical analysis

Descriptive results are presented as mean and standard deviation. The Shapiro-Wilk normality test and test for homogeneity of variances were performed for all data. Two-way analysis of variance for repeated measures with Bonferroni correction was employed to compare pre- and postoperative values. Pearson’s Chi-squared test or the Kruskal-Wallis test was used to compare differences between groups. A bivariate normal analysis was performed prior to the correlation test. Pearson’s or Spearman’s correlation tests were used to determine the association between changes in posterior corneal elevation and lenticule thickness. A stepwise multiple linear regression model was performed to explore possible factors affecting posterior corneal elevation changes, such as lenticule thickness, lenticule refractive power, preoperative corneal characteristics (corneal thickness, Q value, K value in anterior corneal surface and posterior corneal surface, total corneal refractive power). Statistical analyses were performed using SPSS (version 20.0; SPSS Inc., Chicago, IL, USA). Statistical significance was set at *P* < 0.05.

## Results

Eleven eyes of 9 patients in the SMI-LIKE group and 12 eyes of 9 patients in the FS-LIKE group were included. All surgeries were completed successfully and no complications occurred either during or after the procedure. At each follow-up visit, the lenticules were transparent and exhibited good centration and visible demarcation lines. No eyes lost ≥ 2 lines of visual acuity in any group. The mean postoperative SE was − 0.68 ± 1.02 diopter (D) (range − 2.75 to 1.13 D) and − 0.04 ± 0.85 D (range − 1.50 to 1.25 D), in the SMI-LIKE and FS-LIKE groups, respectively. Details are presented in Table 1.

**Table 1:** Patient demographics

Parameter		Mean ± standard deviation (range)		
		SMI-LIKE	FS-LIKE	*P* value
Preoperative				
	Age (years)	24.3 ± 6.6 (18–36)	23.5 ± 5.4 (18–36)	0.976
	Number of eyes (male/female)	7/4	9/3	0.667
	Sphere (D)	5.91 ± 1.36 (4.00–7.75)	6.63 ± 2.57 (3.00–11.00)	0.419
	Cylinder (D)	− 0.98 ± 0.33 (− 0.50– − 1.50)	− 1.06 ± 0.87 (− 2.75–0.00)	0.762
	SE (D)	5.42 ± 1.40 (3.50–7.38)	6.10 ± 2.66 (2.75–11.00)	0.462
	UDVA (logMAR)	0.55 ± 0.36 (0.20–1.20)	0.63 ± 0.34 (0.30–1.20)	0.402
	CDVA (logMAR)	0.28 ± 0.31 (0.00–1.00)	0.30 ± 0.28 (0.10–1.00)	0.423
Implanted lenticule	Thickness (µm)	115.45 ± 22.91 (81–147)	106.58 ± 29.14 (65–152)	0.429
	Optical zone (mm)	6.64 ± 0.11 (6.5–6.8)	6.50	
Postoperative				
	Sphere (D)	0.02 ± 1.08 (− 1.75–1.75)	0.71 ± 0.77 (0.00–2.25)	0.637
	Cylinder (D)	− 1.41 ± 0.96 (− 3.50– − 0.50)	− 1.50 ± 0.94 (− 0.75–3.25)	0.884
	SE (D)	− 0.68 ± 1.07 (− 2.75–1.13)	− 0.04 ± 0.89 (− 1.50–1.25)	0.657
	UDVA (logMAR)	0.35 ± 0.28 (0.10–1.00)	0.39 ± 0.24 (0.20–1.00)	0.290
	CDVA (logMAR)	0.25 ± 0.28 (0.00–1.00)	0.30 ± 0.27 (0.10–1.00)	0.294

*SMI-LIKE* = small-incision lenticule intrastromal keratoplasty; *FS-LIKE* = femtosecond laser-assisted lenticule intrastromal keratoplasty; *D* = diopters; *SE* = spherical equivalent; *UDVA* = uncorrected distance visual acuity; *CDVA* = corrected distance visual acuity; *logMAR* = logarithm of the minimum angle of resolution

In the SMI-LIKE group, PCE and PME-2 mm showed a backward change at one day postoperatively and gradually returned to baseline at one month. Conversely, in the FS-LIKE group, these two variables exhibited forward displacement with fluctuation during the three-month observation after the procedure, and the peak anterior changes were reached at one day postoperatively. Between 1 and 3 months postoperatively, PCE and PME-2 mm showed no apparent change in both groups.

For peripheral regions, PME-6 mm showed forward displacement at one day postoperatively and backward change at one month in the SMI-LIKE group (*P* = 0.090). In the FS-LIKE group, PME-6 mm showed apparent backward change at one day postoperatively, and a gradual backward trend at one month postoperatively (*P* = 0.016).

As for mid-peripheral regions, PCE-4 mm and PME-4 mm exhibited slightly backward change after lenticule implantation in both groups. For PME-4 mm, the backward change at one day postoperatively in the FS-LIKE group was more remarkable than that in the SMI-LIKE group, and a significant difference was noted in the FS-LIKE group (*P* = 0.028). From one day to three months postoperatively, PME-4 mm continuously displayed backward trend in the SMI-LIKE group; but in the FS-LIKE group, it displayed a minuscule forward alteration. No significant differences were found between implant lenticule thickness and changes in posterior elevation in both groups. No association was detected between preoperative corneal thickness, lenticule refractive power or other factors with respect to posterior corneal changes.

In the SMI-LIKE group, Kmb exhibited flattening at one month and subsequently returned to its original level at three months postoperatively; however, no significant statistical difference was found in Kmb through the three-month observation. In the FS-LIKE group, Kmb steepened after lenticule implantation, with a significant change occurring at one day postoperatively (*P* = 0.001).

**Fig. 3:**
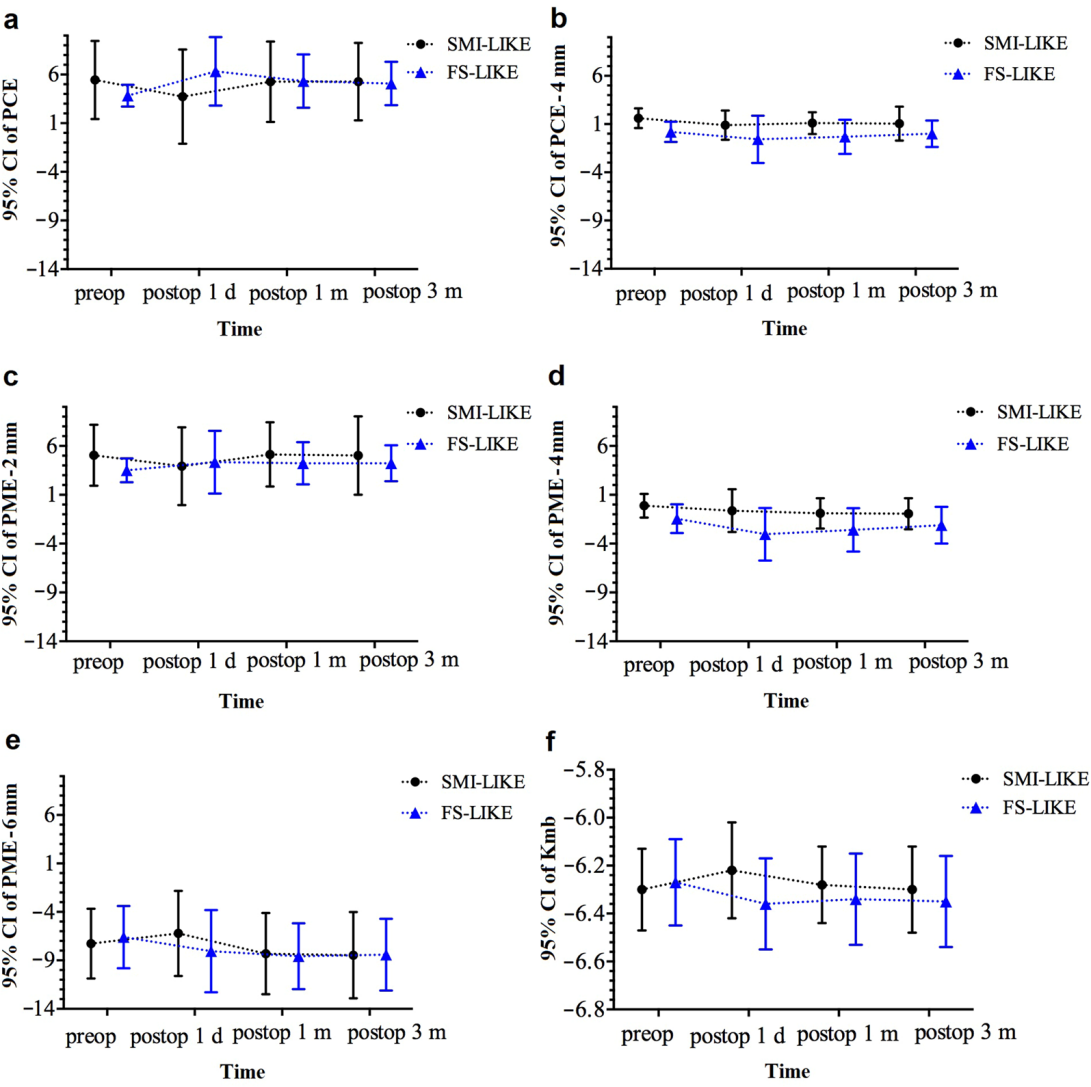
Posterior corneal elevation and mean keratometry of the posterior corneal surface (Kmb) in both two groups at different times. **a** Posterior central elevation (PCE); **b** Mean posterior corneal elevation in the central 4-mm zone of 13 points (PCE-4 mm); **c** Mean posterior corneal elevation in 2-mm optical zone of 4 points (PME-2 mm); **d** Mean posterior corneal elevation in 4-mm optical zone of 8 points (PME-4 mm); **e** Mean posterior corneal elevation in 6-mm optical zone of 14 points (PME-6 mm); **f** Kmb. SMI-LIKE, small-incision lenticule intrastromal keratoplasty; FS-LIKE, femtosecond laser-assisted lenticule intrastromal keratoplasty

**Fig. 4:**
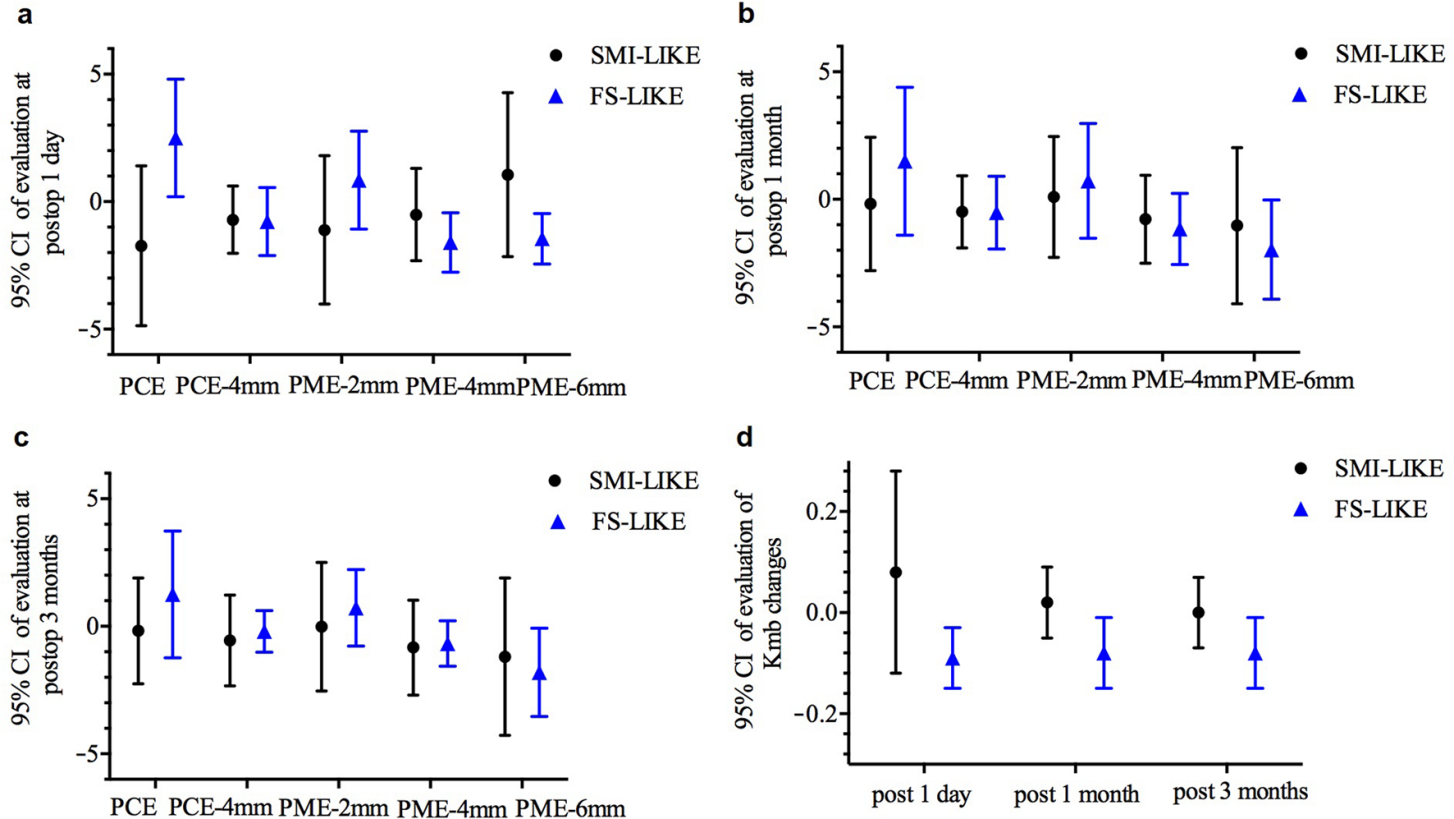
Changes in posterior corneal elevation and keratometry of the posterior corneal surface in both two groups at different times. **a** Changes in posterior corneal elevation at 1 day postoperatively; **b** Changes in posterior corneal elevation at 1 month postoperatively; **c** Changes in posterior corneal elevation at 3 months postoperatively; **d** Kmb changes of different visit times. PCE, posterior central elevation; PCE-4 mm, mean posterior corneal elevation in the central 4-mm zone of 13 points; PME-2 mm, mean posterior corneal elevation in 2-mm optical zone of 4 points; PME-4 mm, mean posterior corneal elevation in 4-mm optical zone of 8 points; PME-6 mm, mean posterior corneal elevation in 6-mm optical zone of 14 points; Kmb, mean keratometry of the posterior corneal surface; SMI-LIKE, small-incision lenticule intrastromal keratoplasty; FS-LIKE, femtosecond laser-assisted lenticule intrastromal keratoplasty

**Fig. 5:**
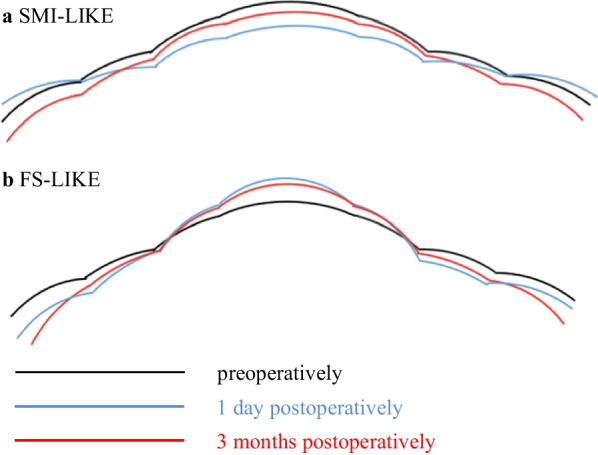
Summary of the posterior corneal elevation in terms of forward or backward change centrally and peripherally for both procedures. Black lines, blue lines and red lines indicate posterior corneal elevation preoperatively, at 1 day and 3 months postoperatively, respectively. **a** Posterior corneal elevation changes in SMI-LIKE; **b** Posterior corneal elevation changes in FS-LIKE. SMI-LIKE, small-incision lenticule intrastromal keratoplasty; FS-LIKE, femtosecond laser-assisted lenticule intrastromal keratoplasty

The posterior corneal elevation data and Kmb values of each visit are presented in Table 2. Table 3 summarizes the changes in posterior corneal elevation and Kmb at different times and the comparison between groups. Figures 3 and 4 illustrate the trends of all values in both groups. Figure 5 is a summary diagram of the posterior corneal elevation in terms of forward or backward change centrally and peripherally for both procedures.

**Table 2:** Posterior corneal elevation (μm) and Kmb before and after femtosecond allogenic lenticule implantation

Parameter	Time point				*P* value			
	Preop	Postop 1 d	Postop 1 m	Postop 3 m		Preop-postop 1 d	Preop-postop 1 m	Preop-postop 3 m
SMI-LIKE								
PCE	5.45 ± 4.01	3.73 ± 4.84	5.27 ± 4.13	5.27 ± 3.98	0.139	–	–	–
PCE-4 mm	1.61 ± 1.02	0.89 ± 1.52	1.11 ± 1.13	1.05 ± 1.76	0.523	–	–	–
PME-2 mm	5.05 ± 3.12	3.93 ± 3.98	5.14 ± 3.29	5.02 ± 4.01	0.417	–	–	–
PME-4 mm	− 0.12 ± 1.21	− 0.63 ± 2.19	− 0.90 ± 1.55	− 0.94 ± 1.58	0.408	–	–	–
PME-6 mm	− 7.27 ± 3.60	− 6.22 ± 4.39	− 8.30 ± 4.20	− 8.46 ± 4.45	0.090	–	–	–
Kmb	− 6.30 ± 0.17	− 6.22 ± 0.20	− 6.28 ± 0.16	− 6.30 ± 0.18	0.259	–	–	–
FS-LIKE								
PCE	3.83 ± 1.11	6.33 ± 3.52	5.33 ± 2.74	5.08 ± 2.23	0.124	–	–	–
PCE-4 mm	0.20 ± 1.05	− 0.58 ± 2.45	− 0.32 ± 1.77	0.00 ± 1.37	0.479	–	–	–
PME-2 mm	3.51 ± 1.21	4.35 ± 3.21	4.23 ± 2.16	4.23 ± 1.84	0.618	–	–	–
PME-4 mm	− 1.44 ± 1.47	− 3.04 ± 2.70	− 2.59 ± 2.22	− 2.12 ± 1.88	0.028	0.016	–	–
PME-6 mm	− 6.60 ± 3.21	− 8.05 ± 4.24	− 8.57 ± 3.39	− 8.41 ± 3.69	0.016	–	0.038	0.032
Kmb	− 6.27 ± 0.18	− 6.36 ± 0.19	− 6.34 ± 0.19	− 6.35 ± 0.19	0.001	0.027	–	–

*SMI-LIKE* = small-incision lenticule intrastromal keratoplasty;* FS-LIKE* = femtosecond laser-assisted lenticule intrastromal keratoplasty; *Kmb* = mean keratometry of the posterior corneal surface; *Preop* = preoperative; *Postop* = postoperative; *d* = day; *m* = month; *PCE* = posterior central elevation; *PCE-4 mm* = mean posterior corneal elevation in the central 4-mm zone of 13 points; *PME-2 mm* = mean posterior corneal elevation in the 2-mm optical zone as a function of the meridian of 4 points; *PME-4 mm* = mean posterior corneal elevation in the 4-mm optical zone as a function of the meridian of 8 points; *PME-6 mm* = mean posterior corneal elevation in the 6-mm optical zone as a function of the meridian of 14 points; *Preop-postop 1 d* = *P* value between preoperatively and 1 day postoperatively; *Preop-postop 1 m* = *P* value between preoperatively and 1 month postoperatively; *Preop-postop 3 months* = *P* value between preoperatively and 3 months postoperatively

**Table 3:** Changes of posterior corneal elevation and Kmb after femtosecond allogenic lenticule implantation

Parameter	Postop 1 d			Postop 1 m			Postop 3 m		
	SMI-LIKE	FS-LIKE	*P* value	SMI-LIKE	FS-LIKE	*P* value	SMI-LIKE	FS-LIKE	*P* value
PCE	− 1.73 ± 3.29	2.50 ± 3.61	0.007	− 0.18 ± 2.75	1.50 ± 3.03	0.149	− 0.18 ± 2.18	1.25 ± 2.60	0.098
PCE-4 mm	− 0.71 ± 1.38	− 0.78 ± 2.50	0.910	− 0.49 ± 1.48	− 0.52 ± 1.48	0.794	− 0.56 ± 1.87	− 0.20 ± 0.84	0.623
PME-2 mm	− 1.11 ± 3.05	0.84 ± 3.08	0.135	0.09 ± 2.49	0.72 ± 2.35	0.467	− 0.02 ± 2.65	0.72 ± 1.57	0.328
PME-4 mm	− 0.51 ± 1.90	− 1.60 ± 2.47	0.226	− 0.78 ± 1.82	− 1.16 ± 1.47	0.363	− 0.83 ± 1.95	− 0.68 ± 0.93	0.974
PME-6 mm	1.06 ± 3.36	− 1.45 ± 2.19	0.067	− 1.03 ± 3.21	− 1.97 ± 2.03	0.265	− 1.19 ± 3.24	− 1.81 ± 1.81	0.343
Kmb	0.08 ± 0.20	− 0.09 ± 0.06	0.013	0.02 ± 0.07	− 0.08 ± 0.07	0.004	0.00 ± 0.07	− 0.08 ± 0.07	0.006

*SMI-LIKE* = small-incision lenticule intrastromal keratoplasty;* FS-LIKE* = femtosecond laser-assisted lenticule intrastromal keratoplasty; *Kmb* = mean keratometry of the posterior corneal surface; *Postop* = postoperative; *d* = day; *m* = month; *PCE* = posterior central elevation; *PCE-4 mm* = mean posterior corneal elevation in the central 4-mm zone of 13 points; *PME-2 mm* = mean posterior corneal elevation in the 2-mm optical zone as a function of the meridian of 4 points; *PME-4 mm* = mean posterior corneal elevation in the 4-mm optical zone as a function of the meridian of 8 points; *PME-6 mm* = mean posterior corneal elevation in the 6-mm optical zone as a function of the meridian of 14 points

## Discussion

Two types of surgical procedures for femtosecond laser-assisted lenticule implantation for hyperopia have been recently introduced in clinical practice: SMI-LIKE and FS-LIKE. To reduce postoperative regression and obtain good optical results, the exact changes in the postoperative corneal surface using these two methods need to be understood. In 2013, Pradhan et al. described a case in which SMI-LIKE was performed to correct for hyperopia [5]. By postoperative day one, there was a significant backward bulge into the anterior chamber on the posterior corneal surface map. After six months, the residual SE refraction was still + 6.00 D and the backward protrusion was also apparent. The authors inferred that posterior corneal surface changes contribute to under-correction. Subsequently, Sun et al. reported five cases of autologous FS-LIKE at a one-year follow-up observation. The representative posterior corneal elevation maps were virtually identical at all time points [6]. Liu and colleagues studied the safety and tissue response of SMI-LIKE in animal models and found no significant changes in posterior corneal elevation after SMI-LIKE compared to preoperative levels [12]. The previously reported posterior corneal surface changes after SMI-LIKE and FS-LIKE are inconsistent. Herein, we report for the first time posterior corneal elevation changes after using both techniques.

Despite the few studies on posterior corneal elevation or corneal biomechanical property changes after lenticule implantation, similar studies after SMILE and FS-LASIK are useful for comparison. Based on the available literature, the differences in biomechanical properties after SMILE and LASIK could be divided into the following: (1) The anterior 40% of cornea fibers are tightly arranged and contribute the most of the corneal tensile strength [13, 14]. Small side-cut in SMILE leaves the anterior corneal lamellae intact, resulting in acceptable corneal biomechanical stability [15]. (2) Bowman’s layer, which possesses stronger biomechanical properties than the corneal stromal, remains intact after SMILE and further maintains corneal stability [16]. (3) Although the LASIK flap is repositioned and healed, the corneal stroma in the flap rarely provides biomechanical strength postoperatively and should be neglected [17]. (4) Moreover, in an established geometric analog model comparing corneal morphology changes after SMILE and LASIK, posterior corneal stress distribution in the residual stromal bed increased after LASIK, but was similar to that observed preoperatively after SMILE [18].

In this study, PCE and PME-2 mm, two calculated values representing posterior corneal changes in the central part of the corneal, showed opposite results at one day after implantation in the two intervention groups: there was a slight decrease with SMI-LIKE and an increase with FS-LIKE. The change pattern in Kmb was consistent with posterior corneal elevation changes in the central part of the corneal: eyes in FS-LIKE steepened with a statistical difference at one day postoperatively while in SMI-LIKE exhibited flattening. The differences between groups can be partially explained by the aforementioned findings for SMILE/LASIK. In SMI-LIKE, most anterior cornea fibers remained intact except for those running across the side cut; whereas in FS-LIKE these strong corneal fibers were excised in the process of scanning and separation. The implanted lenticule exerted expansion forces both for the anterior and posterior parts of the cornea. Corneal stromal lamellae located ahead of the scanning interface were preserved and offered a high tensile strength after SMI-LIKE, leading to a resistance force pushing against the lenticule from the cap; thus, the posterior corneal surface retreated, and the posterior elevation decreased. In contrast, after FS-LIKE, the anterior portion of the cornea was weakened, and little tensile strength was observed. The lenticule experienced little resistance and no backward change in elevation was noted [10]. The results of the current study are consistent with those of Wu and colleagues, who described the outcomes of hyperopia correction using FS-LIKE, and found no anteroposterior forces exerted by lenticule implantation in FS-LIKE [11].

Of note, in Pradhan et al.’s SMI-LIKE case, the patient experienced an apparent central bulge into the anterior chamber at one day postoperatively [5], and posterior corneal elevation decreased significantly from − 2 to − 38 µm after implantation. Studer et al. applied a SMI-LIKE model with an implant depth of 180 µm, and discovered the implanted lenticule led to central posterior corneal surface retreating and backward bulging into the anterior chamber; they also suggested that the thicker the myopic lenticule implanted, the more flattened the posterior corneal surface [9]. One report proposed that the retention of Bowman’s layer resulted in this phenomenon [19]. Although Bowman’s layer was preserved in our SMI-LIKE cases, no such phenomena of inward bulging was found. Furthermore, there was no significant correlation between posterior elevation changes and lenticule thickness. In our study, the implant depth of SMI-LIKE was set at 100 µm, much shallower than that in the aforementioned case (180 µm). Four cases were implanted with lenticule thickness > 130 µm, and none developed backward bulging postoperatively.

Implant depth has become a point of debate in the lenticule keratoplasty technique. Pioneer cases of implant depth were relatively deep (180 µm and 160 µm). In later studies, the implant depth was shallow (i.e., 110 µm and 100 µm). Implant depth along with thickness of the implanted lenticule are considered factors determining the ultimate corneal refractive power. As allogenic lenticules cause complex wound healing responses and may allow tissue integration, the proper implant depth of SMILE-derived lenticules is essential. Studies have reported that thinner corneal flaps/caps may induce more inflammatory cytokines; a thin flap in the FS-LIKE procedure has other potential complications, such as high rates of flap necrosis and epithelial ingrowth [1]. Nevertheless, some researchers still recommend implanting the lenticule at a shallow depth for the following advantages: firstly, a shallow implant depth is associated with a greater percentage of intended correction and less posterior corneal flattening [20]; secondly, it is favorable to have residual myopic diopters because surgical correction of myopia has better stability and efficacy than correction of hyperopia [8]; lastly, overcorrection of hyperopia is acceptable for both patients and surgeons [8]. More studies comparing different implant depths are required to improve the surgical design in the future.

Changes in elevation in the two groups at each timepoint are displayed in Table 3, with the only notable difference occurring in PCE at one day postoperatively. No statistically significant differences were found from one day to three months postoperatively. It has been demonstrated that the corneal wound healing response affects corneal surface stability and changes in posterior corneal elevation [21, 22]. According to the results of animal model studies and clinical practice of FS-LIKE, mild to moderate edema was observed in both the lenticule and the surrounding cornea area on postoperative day one, which then considerably reduced on the second day after FS-LIKE [1, 5, 23]. Similarly, patients who underwent SMI-LIKE experienced such a recovery process [5]. For this reason, it is understandable that the most distinct change was noticed at one day postoperatively. The high-resolution anterior segment optical coherence tomography showed that lenticule edema was the most prominent at the thickest part in SMI-LIKE [24]. The findings provided explanation for our result: after SMI-LIKE, lenticule edema caused posterior corneal surface backward change in the central area and forward displacement in the periphery; as the edema gradually subsided, the posterior corneal surface recovered over time.

This study has several limitations. First, the sample size was small, which may limit the accuracy of the results. This is because of the much smaller number of hyperopia than myopia patients in Asia. As a new procedure for surgical hyperopia correction, the long-term safety and efficacy of allogenic lenticule implantation has not been proven. Few patients met the inclusion criteria and underwent surgery after comprehensive explanation. Second, the follow-up time was restricted to three months. A longer observation of the current cohort is still underway. Third, the small sample size limited us from dividing the patients into subgroups by lenticule thickness or implant depth, which may influence postoperative effects.

## Conclusions

In conclusion, we demonstrated that both SMI-LIKE and FS-LIKE are feasible surgical management strategies for the correction of hyperopia. Posterior corneal surface after SMI-LIKE and FS-LIKE exhibited different change pattern in various corneal regions, with the most prominent change occurring at one day postoperatively during the three-month follow-up. The current study sets the stage for further investigations on the long-term changes in the posterior corneal surface and the proper implant depth for femtosecond laser-assisted lenticule keratoplasty.

## Data Availability

The authors confirm that the data supporting the findings of this study are available within the article and its supplementary materials.

## Abbreviations

BFS: Best-fit-sphere
CDVA: Corrected distance visual acuity
Kmb: Mean keratometry of the posterior corneal surface
FS-LIKE: Femtosecond laser-assisted lenticule intrastromal keratoplasty
LASIK: Laser in situ keratomileuses
PCE: Posterior central elevation
PME: Posterior mean elevation
SE: Spherical equivalent
SMILE: Small incision lenticule extraction
SMI-LIKE: Small-incision lenticule intrastromal keratoplasty
UDVA: Uncorrected distance visual acuity
